# The Effect of Long-Term Exposure to O_3_ and PM_2.5_ on Allergies and Asthma in Adolescents and Young Adults

**DOI:** 10.3390/ijerph22081262

**Published:** 2025-08-12

**Authors:** Aliaksandr Amialchuk, Onur Sapci

**Affiliations:** Department of Economics, University of Toledo, 2801 W. Bancroft St. MS 922, Toledo, OH 43606, USA; onur.sapci@utoledo.edu

**Keywords:** pollution, ozone, particulate matter 2.5, respiratory conditions, Add Health

## Abstract

Using data on the children of the respondents who participated in Wave IV (2008) and Wave V (2016–2018) of the National Longitudinal Study of Adolescent to Adult Health, we estimate the effect of long-term exposure to ozone (O_3_) and particulate matter 2.5 (PM_2.5_) on diagnoses of allergies and asthma in adolescence and young adulthood. Estimates from individual-level fixed-effect models with time-varying controls show that exposure to PM_2.5_ and O_3_ is associated with higher likelihood of asthma and allergies in females at younger ages (10–12 years old) and allergies in males at older ages (13 years old and above). These findings are novel and contribute to the growing body of literature exploring gender and age differences in susceptibility to asthma and allergies.

## 1. Introduction

A lot of controversy surrounds pollution regulation due to the ongoing debate about costs and benefits of such regulations, especially regarding the health benefits. In the absence of experimental data, most of the estimates of health effects of pollution rely on observational data. Several studies published in epidemiological and medical journals establish statistical associations between air pollution and human health. This literature showed that exposure to ambient air pollution is associated with a wide range of health problems [1,2,3,4]. Furthermore, it was suggested that long-term exposure to air pollution early in life may lead to the development of major risk factors for cardiovascular disease, including obesity, hypertension, and metabolic disorders [5], as well as asthma and other respiratory illnesses [6]. Economic studies contributed new data and empirical approaches to identify and estimate the causal effect of air pollution exposure on health by better controlling for unobserved factors that confound the relationship between air pollution and health. Several studies have attempted to identify the causal effect of pollution exposure on health outcomes such as infant mortality, morbidity, hospitalization, and child health [5,7,8,9,10,11].

It is widely recognized that exposure to O_3_ and particulate matter is linked to cardiovascular and respiratory health problems [12,13,14,15]. Furthermore, these effects can be observed even at concentrations below the National Ambient Air Quality Standards [16]. The ground-level O_3_ layer is formed by chemical reactions that occur between volatile organic chemicals and oxides of nitrogen in the presence of sunlight and heat [17]. Primary sources of ground-level O_3_ are vehicle emissions, gasoline vapors, and industrial facilities including power plants [18]. O_3_ affects respiratory morbidity by irritating lung airways, decreasing lung function, and increasing respiratory symptoms [2]. Studies have consistently linked higher O_3_ concentrations with increased health care visits for respiratory diseases (see, e.g., [10]). Breathing O_3_ deeply can trigger a variety of health problems such as shortness of breath, coughing, and chest pain [17]. It can also aggravate asthma, emphysema, chronic bronchitis, and other respiratory diseases (see, e.g., [10]).

Fine particulate matter (PM_2.5_), defined as particles with a diameter of 2.5 µm or less, is primarily emitted by sources such as power plants, industrial activities, and motor vehicles [3]. Because of their small size, these particles can travel deep into the respiratory tract and enter the bloodstream [19]. Both toxicological and epidemiological studies have shown that PM_2.5_ poses significant health risks, particularly to the respiratory and cardiovascular systems [20,21,22]. High levels of short-term exposure can worsen asthma, contribute to lung conditions, and aggravate preexisting heart problems. Long-term exposure has been linked to impaired lung function and an increased risk of premature mortality [23].

In this study we estimate the effect of long-term O_3_ and PM_2.5_ exposure on the diagnoses of asthma and allergies in males and females during early adolescence (ages 10–12), adolescence (ages 13–17), and early adulthood (ages 18–25). Exposure to air pollution during early developmental stages may pose critical risk to health [24,25]. In fact, asthma and allergies are distinct conditions, though they can overlap (allergic asthma). Allergies involve an overreaction of the immune system to substances (allergens) like pollen, pet dander, or dust mites, while asthma is a respiratory condition characterized by airway (bronchial tube) inflammation, making it difficult to breathe [3]. The American Lung Association lists the causes of asthma as family history (genetic predisposition, atopy), allergens, viral respiratory infections, occupational exposures, smoking, air pollution and irritants and obesity (American Lung Association https://www.lung.org/ (accessed on 8 May 2025)).

Medical literature also acknowledges gender differences in asthma and allergy occurrences [26,27,28] but the impact of PM_2.5_ and O_3_ pollution exposure on respiratory symptoms in adolescence is less known. It is generally accepted that boys have a higher prevalence of asthma and allergic diseases than girls in childhood, but this pattern reverses around puberty [28,29]; however, these studies are not specific to pollution exposure. Sex-specific patterns in asthma and allergy may be driven by differences in lung development, hormonal changes during puberty, immune system function, and exposure timing [28,29].

The objective of our study is to quantify the effect of air pollution exposure on different genders and age categories, which is an ongoing research area with no clear consensus. The data that we use also makes it possible to estimate the effect of long-term air pollution exposure by measuring pollution exposure over several years. We also contribute to the literature by using more refined measures of pollution exposure, which are assigned to the respondents based on census tract of residence in each wave of data. In order to identify the causal effect of pollution exposure, we estimate individual fixed-effect models that account for several important time-varying covariates and identify the effect of long-term exposure to O_3_ and PM_2.5_ on asthma and allergies in adolescence and early adulthood.

## 2. Methods

This study draws on data from the National Longitudinal Study of Adolescent to Adult Health (Add Health), a nationally representative, school-based survey that began in 1994–1995 with approximately 90,000 adolescents enrolled in grades 7 through 12 across 132 schools in the U.S. A representative subset of these students (N = 20,745) participated in in-home interviews during Wave I (1994–1995), with a follow-up conducted in 1995–1996 (Wave II, N = 14,738). A majority of these participants were re-interviewed in subsequent waves—Wave IV in 2008 (N = 15,701) and Wave V in 2016–2018 (N = 12,300)—when they were between the ages of 33 and 43 [30].

Our sample included children of the original respondents who responded to the in-home survey in Waves IV and V of Add Health. In each of these surveys, the respondents were asked to fill out a questionnaire asking about live births where they were asked about their children. After merging the data on children between Waves IV and V based on month/year of birth and gender of child, the resulting sample of children had 7187 individual child records. After further restricting the sample to non-missing responses on the pollution variables (excluded 71 records), non-missing responses on the dependent variables (excluded an additional 12 records), restricting the age of children to be at least one year old in Wave V (excluded an additional 4 records) and restricting the age of children to be between at least 10 years old and at most 25 years old at Wave V interview (excluded additional 240 records), the sample used in our analysis consisted of 6860 children observed in both waves. All of the analyses utilized Wave IV-V longitudinal sampling weights. (Add Health uses a multistage clustered sample design with observations that have unequal probability of selection, and requires the use of sampling weights to make the estimates nationally representative [31].)

### 2.1. Pollution Exposure Variables

The annual average pollutant concentrations used in this study were generated by Add Health researchers using publicly available daily census-tract-level estimates from the U.S. Environmental Protection Agency’s Fused Air Quality Surface using Downscaling (FAQSD) data. The FAQSD approach integrates data from ground-based air quality monitors with outputs from atmospheric models to produce high-resolution pollution estimates. These estimates are derived using a Bayesian space–time downscaling model, which applies advanced statistical techniques to create detailed, fused predictions of air quality over time and space. For each calendar year from 2002 to 2017, daily PM_2.5_ estimates were averaged to produce annual concentration levels [32].

The same procedure was used to create annual average census-tract-level O_3_ concentration estimates. Annual census-tract-level averages were assigned to each respondent based on the census tract of residence in each wave of Add Health. Estimated pollutant concentrations in 2002–2005 were assigned to respondents based on their census tract of residence reported at Wave III (2002); estimated annual pollutant concentrations in 2006–2011 were assigned to respondents based on census tract of residence reported at Wave IV; and annual pollutant concentrations in 2012–2017 were assigned based on census tract of residence reported at Wave V. For PM_2.5_ the units are micrograms of PM_2.5_ per cubic meter of air (µg/m^3^) and for O_3_ the units are parts per billion (ppb). For each pollutant, yearly values were averaged across years 2002–2008 if the child was born before 2002 and were averaged for the years since the year of birth until 2008, and then these averages were assigned as Wave IV pollution measures. Yearly pollutant values were averaged across years 2009–2016 if the year of Wave V interview was in 2016 and averaged across years 2009–2017 if the year of Wave V interview was 2017 or later, and then these averages were assigned as Wave V pollution measures.

### 2.2. Health Outcomes

Add Health respondents were asked a series of questions about the health of their children in Wave IV and Wave V. Our analysis focuses on respiratory conditions and uses two binary dependent variables: allergy and asthma. (We did not use the question asking about “Any other chronic respiratory, lung, or breathing condition” in children because only about 1% of the children were confirmed to have ever been diagnosed with these.) The indicator for allergies was set equal to one if the respondent answered affirmatively to the question “Has a doctor ever told you that {fill child’s name} has any of these conditions? Allergies or hay fever, not including allergic reactions to medications”. The indicator for asthma was set equal to one if the respondent answered affirmatively to the question “Has a doctor ever told you that {fill child’s name} has any of these conditions? Asthma.”

### 2.3. Other Variables

Several studies previously incorporated measures of socioeconomic status such as unemployment, education, occupational class, and income when analyzing respiratory problems [33,34,35,36,37,38]. For example, several studies found that children from affluent families (The Hygiene Hypothesis) tend to be diagnosed with allergies more often [39]. Therefore, our multivariate analysis includes several relevant time-varying controls measured in both Waves IV and V: a variable “Household size” measured as the number of people reported to be residing in the household not including the respondent; an indicator variable “Parent has college degree” equal to one if the responding parent (usually the mother) has a college degree; an indicator variable “Parent works for pay” equal to one if the responding parent is currently working for pay; a variable “Parent work hours” measuring total hours a week that the responding parent usually spends at all her jobs; a categorical variable “Household income bracket” indicating total household income bracket from 1 (less than USD 5000) to 12 (USD 150,000 or more) in increments of USD 5000; an indicator variable “Parental employment HI” equal to one if the respondent’s employer makes health insurance available to them; a variable “Parent smoking days” measuring on how many days the respondent smoked cigarettes during the past 30 days; and a variable “Parent marijuana use days” measuring on how many days the respondent used marijuana during the past 30 days. We stratify our analysis by age and gender of the child.

Table 1 displays sample summary statistics for each wave. The incidence of allergies increased from 9.4% in Wave IV when the children were about 6 years old to 15.7% in Wave V when they were about 15 years old. The corresponding incidence of asthma increased from 10.2% to 13.6%. During the same period, average O_3_ concentration decreased from 39.06 to 38.79 ppb and PM_2.5_ concentration decreased from 12.17 to 9.47 µg/m^3^.

## 3. Empirical Model and Estimation Strategy

We estimate the following regression model:

(1) $${Prob(Y}_{it}=1)=\alpha +\beta_{1}{O_{3}}_{it}+\beta_{2}{{PM}_{2.5}}_{it}+\beta_{3}X_{it}+\delta_{i}+\gamma_{t}+\varepsilon_{it}$$

where $Y_{it}$ is the outcome variable (indicators for having been diagnosed with allergy or asthma) measured in Wave IV and Wave V, ${O_{3}}_{it}$ is the measure of the O_3_ pollution exposure, ${{PM}_{2.5}}_{it}$ is the measure of PM_2.5_ pollution exposure, $X_{ist}$ is a vector of household and parental control variables,$\;\delta_{i}$ is the child-specific fixed effect, and $\varepsilon_{ist}$ is the error term. The model also includes child-specific fixed effects, $\delta_{i}$, which control for any unobserved fixed child-specific factors that may be related to both the respiratory problems and the pollution exposure variables. $\gamma_{t}$ is the time fixed effect captured by Wave 5 dummy. We estimate Equation (1) using a linear probability model and the coefficients $\beta_{1}$ and $\beta_{2}$ represent the effect of a one-unit increase in long-term average pollution exposure (one more ppb for O_3_, one more µg/m^3^ for PM_2.5_) on the probability of ever being diagnosed with each respiratory outcome.

Identification of the effect of pollution on the probability of the health conditions in Equation (1) relies on conditional independence assumption: after using child-specific fixed effects, $\delta_{i}$, general time trend, $\gamma_{t}$, and household and parent-specific time-varying controls, $X_{ist},$ all of which are meant to account for differences in demographic and socio-economic status across and within families over time that may be related to the choice of residential location by the families, the remaining variation in residence-specific pollution exposure across and within families is exogenous and does not depend on the potential respiratory outcomes of the children.

## 4. Results

**Figure 1:**
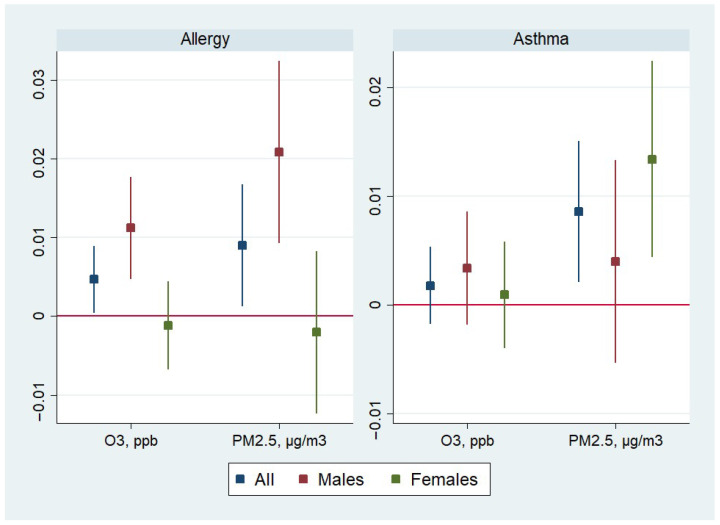
Estimates (with 95% confidence intervals) of the effect of pollution on respiratory problems from linear models with child fixed effects, by gender.

Table 2 reports the estimated effects of O_3_ and PM_2.5_ pollution exposure on allergies and asthma from the fixed-effect regression and Figure 1 illustrates these estimates. The estimates are reported for the full sample and stratified by gender. In the full sample, both pollutants have a significant positive effect on the probability of ever being diagnosed with allergies: an increase in O_3_ by 1 ppb increases the likelihood of being diagnosed with allergies by 0.5 percentage points (pp); an increase in PM_2.5_ by one µg/m^3^ increases the likelihood of being diagnosed with allergies by 0.9 pp. Put differently, these estimates imply that a decrease in O_3_ and PM_2.5_ would directly lead to a decrease in allergies and asthma. While the raw tabulations presented in Table 1 show that the prevalence of asthma and allergies was increasing between Waves IV and V at the same time as the concentrations of the pollutants were decreasing, the regression estimates show the expected positive associations between the air pollutants and the respiratory outcomes after these associations were adjusted for the influence of fixed unobservable individual factors as well as the time-varying household and parental covariates. These effects are also present for males with the magnitudes of the effects approximately double. PM_2.5_ significantly increases the likelihood of females ever being diagnosed with asthma: an increase in PM_2.5_ by 1 µg/m^3^ increases the likelihood of asthma diagnosis by 1.3 pp among females.

**Figure 2:**
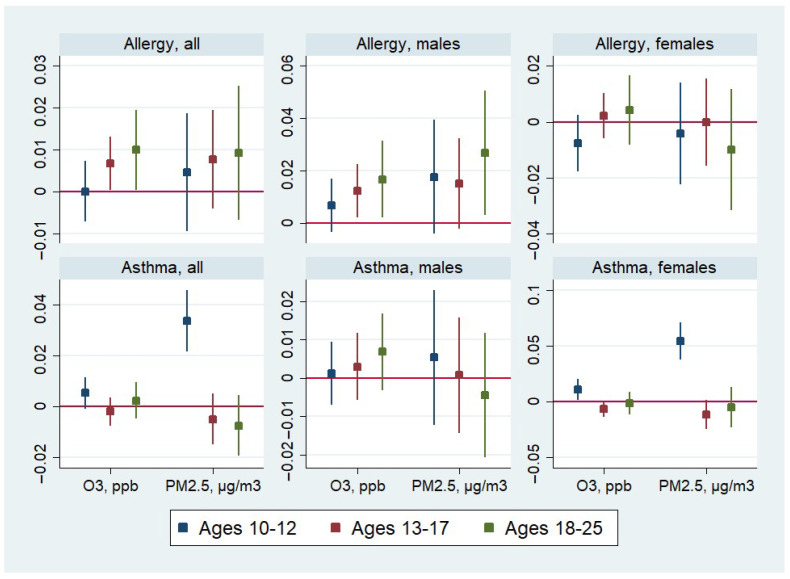
Estimates (with 95% confidence intervals) of the effect of pollution on respiratory problems from linear models with child fixed effects, by gender and age.

Table 3 reports the estimated effects stratified by age groups in addition to gender: early adolescents (ages 10–12 in Wave V), adolescents (ages 13–17 in Wave V) and young adults (ages 18–25 in Wave V). The same estimates and the associated confidence intervals are illustrated in Figure 2. The effects of the pollutants on allergies manifest themselves starting at age 13: exposure to O_3_ significantly increases the likelihood of allergies among adolescents and young adults and exposure to PM_2.5_ significantly increases the likelihood of allergies but only among young adults. These effects are only present among males and the magnitudes of these effects are larger among young adults compared to adolescents. By contrast, only females in early adolescence are affected by the exposure to the pollutants: both O_3_ and PM_2.5_ increase the likelihood of asthma in this sub-group.

## 5. Discussion

According to our results, O_3_ and PM_2.5_ trigger asthma in girls during early adolescence (up to ages 10–12 in Wave V) but these effects fade out as age increases into adolescence and young adulthood. On the other hand, ozone exposure-related allergies start showing significant effects in boys during adolescence (ages 13–17) and these effects intensify in young adulthood (up to ages 18–25). PM_2.5_-related allergic reaction is highly significant for boys again in young adulthood years. Overall, our estimates suggest that pollution exposure during adolescence and young adulthood leads to significant asthma and allergic reaction problems with the effects of the two pollutants varying by gender and by age.

Air pollution may contribute to the development of asthma and allergies through several well-established biological channels. Pollutants like ozone and fine particulate matter can irritate the airways and trigger inflammatory responses, particularly in developing lungs [40]. They may also increase the likelihood of allergic sensitization by weakening the respiratory system’s defenses and altering immune function [41]. Early-life exposure appears especially important, as it can affect both lung development and immune system regulation [42]. There is also growing evidence that long-term exposure can lead to lasting biological changes, including epigenetic modifications, that may increase susceptibility to respiratory conditions later in life [43,44,45].

Differences in how boys and girls respond to air pollution exposure may reflect underlying variation in lung development, immune function, and the timing of biological changes during adolescence [46,47,48]. For instance, girls typically reach lung maturity earlier and may exhibit stronger immune responses during puberty, which could explain greater sensitivity to pollution-related asthma at younger ages. Boys, on the other hand, may be more vulnerable at older ages due to slower physiological development and smaller airway size during childhood. Hormonal shifts during adolescence likely also play a role in shaping these responses [49,50,51]. These patterns are consistent with previous research suggesting that biological, environmental and developmental factors help explain gender and age differences in vulnerability to air pollution (e.g., [28,29]).

Our research provides empirical evidence that the respiratory health impacts of pollution exposure vary by age and gender; however, further medical research is needed to fully understand the underlying biological mechanisms behind these differences. Our findings are consistent with a growing body of evidence highlighting the adverse health effects of ambient air pollution. By stratifying our results by gender and age, we provide more detailed insight into heterogeneous responses to air pollution. The age- and gender-specific nature of our results represents a novel contribution to the literature on air pollution and respiratory health.

Despite the strengths of our empirical strategy, several limitations remain. Potential time-varying confounders including ambient temperature and humidity, endogenous residential mobility, and sample attrition may bias our estimates. In addition, exposure to airborne pollutants is not measured directly but only approximated using annual average pollutant concentrations at the census tract level. As such, we interpret our findings as evidence of strong associations rather than definitive causal effects.

## 6. Conclusions

Combining data on children of respondents from Waves IV (2008) and V (2016–2018) of the National Longitudinal Study of Adolescent to Adult Health with census-tract-level concentration estimates of ozone (O_3_) and fine particulate matter (PM_2.5_) for each respondent, we estimate the impact of long-term exposure to these pollutants on the likelihood of allergy and asthma diagnoses during adolescence and early adulthood. Using child-specific fixed-effect models with time-varying controls, we find that exposure to O_3_ and PM_2.5_ is associated with a higher likelihood of asthma among females at younger ages (10–12 years old), and with a higher likelihood of allergies among males at older ages (13 and above).

This study contributes new evidence on the age- and gender-specific associations between long-term air pollution exposure and respiratory health outcomes in youth. Our stratified estimates highlight the importance of considering developmental timing and biological differences in vulnerability to environmental stressors. By leveraging longitudinal data and a fixed-effect approach, we provide a stronger empirical basis for interpreting these relationships, although we acknowledge limitations such as possible unobserved time-varying confounders and selective attrition.

Our findings have meaningful implications for public health and environmental policy. They suggest that exposure to ambient air pollution may have different health impacts depending on the timing of exposure and the demographic characteristics of the exposed individuals. Interventions targeting pollution reduction and exposure mitigation during critical developmental windows—especially for young girls and adolescent boys—may yield substantial health benefits. Moreover, recognizing gender and age heterogeneity in exposure effects may improve the design of more equitable health and environmental regulations.

Future research should build on this work by exploring causal mechanisms in greater detail, using alternative identification strategies and expanded datasets, measuring pollution exposure more precisely using time spent by the respondents in outdoor activities, exploring underlying biological and environmental mechanisms, and assessing the impact of other outdoor and indoor pollutants. Additional work is also needed to assess whether these associations persist or evolve into later adulthood and whether cumulative exposure has broader effects on long-term health or labor market outcomes. As the health costs of environmental exposures become more apparent, integrating insights from economics, epidemiology, and environmental science will be essential for developing effective and targeted policy responses.

## Figures and Tables

**Table 1:** Sample summary statistics by wave, panel of 6860 children of Add Health respondents.

*Wave IV (2008)*	Mean	SD	Min	Max
Allergy	0.094	0.292	0	1
Asthma	0.102	0.303	0	1
O_3_, ppb	39.06	3.608	24.83	53.72
PM_2.5_, µg/m^3^	12.17	2.269	4.850	18.73
Time-varying covariates:				
Household size	3.421	1.439	1	14
Parent has college degree	0.183	0.386	0	1
Parent works for pay	0.613	0.487	0	1
Parent work hours	0.349	1.550	0	14
Household income bracket	7.441	2.874	1	12
Parental employment HI	0.683	0.466	0	1
Parent smoking days	8.412	12.92	0	30
Parent marijuana use days	0.441	1.381	0	6
Grouping characteristics:				
Age	5.778	3.339	1	16
Male	0.505	0.500	0	1
***Wave V (2016–2018)***				
Allergy	0.157	0.363	0	1
Asthma	0.136	0.343	0	1
O_3_, ppb	38.79	3.100	25.87	53.85
PM_2.5_, µg/m^3^	9.467	1.407	4.717	14.61
Time-varying covariates:				
Household size	4.255	3.831	1	22.60
Parent has college degree	0.245	0.430	0	1
Parent works for pay	0.801	0.399	0	1
Parent work hours	4.259	2.542	0	15
Household income bracket	7.668	3.537	1	12
Parental employment HI	0.617	0.486	0	1
Parent smoking days	7.489	12.57	0	30
Parent marijuana use days	0.704	1.738	0	6
Grouping characteristics:				
Age	14.72	3.398	10	25
Male	0.505	0.500	0	1

**Table 2:** Estimates of the effect of pollution on respiratory problems from linear models with child fixed effects, by gender.

	Allergy, All	Allergy, Males	Allergy, Females	Asthma, All	Asthma, Males	Asthma, Females
O_3_, ppb	0.005 *	0.011 **	−0.001	0.002	0.003	0.001
	(0.002)	(0.003)	(0.003)	(0.002)	(0.003)	(0.003)
PM_2.5_, µg/m^3^	0.009 *	0.021 **	−0.002	0.009 **	0.004	0.013 **
	(0.004)	(0.006)	(0.005)	(0.003)	(0.005)	(0.005)
Household size	−0.000	−0.002	0.001	0.000	0.002	−0.001
	(0.001)	(0.002)	(0.002)	(0.001)	(0.002)	(0.001)
Parent has college degree	0.053 **	0.054 *	0.052 *	0.006	0.018	−0.008
	(0.017)	(0.025)	(0.023)	(0.014)	(0.020)	(0.020)
Parent works for pay	0.003	−0.018	0.027 *	−0.014	0.015	−0.047 **
	(0.010)	(0.014)	(0.013)	(0.008)	(0.011)	(0.011)
Parent work hours	−0.006 **	−0.004	−0.008 **	−0.003	−0.002	−0.003
	(0.002)	(0.003)	(0.002)	(0.002)	(0.002)	(0.002)
Household income bracket	0.001	0.001	0.001	0.001	0.001	0.001
	(0.002)	(0.002)	(0.002)	(0.001)	(0.002)	(0.002)
Parental employment HI	−0.007	−0.006	−0.006	0.014	0.004	0.025 *
	(0.009)	(0.013)	(0.012)	(0.008)	(0.011)	(0.011)
Parent smoking days	−0.000	0.001	−0.001	0.000	0.001	−0.001
	(0.000)	(0.001)	(0.001)	(0.000)	(0.001)	(0.001)
Parent marijuana use days	−0.010 **	−0.012 **	−0.009 *	−0.002	−0.004	−0.001
	(0.003)	(0.004)	(0.004)	(0.002)	(0.003)	(0.004)
Wave 5 dummy	0.108 **	0.143 **	0.076 **	0.065 **	0.037 *	0.094 **
	(0.014)	(0.021)	(0.018)	(0.012)	(0.017)	(0.016)
N. of children	6860	3462	3398	6860	3462	3398

Note: Statistical significance: * *p* < 0.05, ** *p* < 0.01. Standard errors in parentheses. See the text for the definition and units of measurement of each variable.

**Table 3:** Estimates of the effect of pollution on respiratory problems from linear models with child fixed effects, by gender and age.

	Allergy, All	Allergy, Males	Allergy, Females	Asthma, All	Asthma, Males	Asthma, Females
***Age in Wave V: 10–12***						
O_3_, ppb	0.000	0.007	−0.008	0.005	0.001	0.011 *
	(0.00)	(0.01)	(0.01)	(0.00)	(0.00)	(0.00)
PM_2.5_, µg/m^3^	0.005	0.018	−0.004	0.034 **	0.005	0.055 **
	(0.01)	(0.01)	(0.01)	(0.01)	(0.01)	(0.01)
N. of children	2216	1110	1106	2216	1110	1106
***Age in Wave V: 13–17***						
O_3_, ppb	0.007 *	0.012 *	0.002	−0.002	0.003	−0.006
	(0.00)	(0.01)	(0.00)	(0.00)	(0.00)	(0.00)
PM_2.5_, µg/m^3^	0.008	0.015	−0.000	−0.005	0.001	−0.011
	(0.01)	(0.01)	(0.01)	(0.01)	(0.01)	(0.01)
N. of children	3082	1554	1528	3082	1554	1528
***Age in Wave V: 18–25***						
O_3_, ppb	0.010 *	0.017 *	0.004	0.002	0.007	−0.001
	(0.00)	(0.01)	(0.01)	(0.00)	(0.01)	(0.01)
PM_2.5_, µg/m^3^	0.009	0.027 *	−0.010	−0.007	−0.005	−0.005
	(0.01)	(0.01)	(0.01)	(0.01)	(0.01)	(0.01)
N. of children	1562	798	764	1562	798	764

Note: Statistical significance: * *p* < 0.05, ** *p* < 0.01. Standard errors in parentheses. See the text for the definition and units of measurement of each variable. All control variables from Table 2 are included in the estimated models.

## Data Availability

This research uses data from Add Health, a program project directed by Kathleen Mullan Harris and designed by J. Richard Udry, Peter S. Bearman, and Kathleen Mullan Harris at the University of North Carolina at Chapel Hill, and funded by grant P01-HD31921 from the Eunice Kennedy Shriver National Institute of Child Health and Human Development, with cooperative funding from 23 other federal agencies and foundations. Special acknowledgment is due for Ronald R. Rindfuss and Barbara Entwisle for assistance in the original design. Information on how to obtain the Add Health data files is available on the Add Health website (http://www.cpc.unc.edu/addhealth) (accessed on 8 May 2025). No direct support was received from grant P01-HD31921 for this analysis. The use of Add Health data by the authors of this manuscript received “Exempt” status by the University of Toledo Institutional Review Board. Information on how to reproduce the analysis is available from the corresponding author on request.
